# Exploring sex-specific causal links between thousands of proteins and lipid metabolism using the UK Biobank Pharma Proteomics Project data

**DOI:** 10.1186/s13293-026-00934-5

**Published:** 2026-06-16

**Authors:** Daniela Zanetti, Mine Koprulu, Federica Grosso, Daria V. Zhernakova, Francesca Crobu, Serena Sanna

**Affiliations:** 1 Institute of Genetic and Biomedical Research (IRGB), National Research Council (CNR), Cagliari, Italy; 2 Precision Healthcare University Research Institute, Queen Mary University of London, London, UK; 3 MRC Epidemiology Unit, School of Clinical Medicine, Institute of Metabolic Science, University of Cambridge, Cambridge, CB2 0QQ UK; 4 Computational Medicine, Berlin Institute of Health at Charité-Universitätsmedizin Berlin, 10117 Berlin, Germany; 5 Department of Genetics, University of Groningen, University Medical Center Groningen, Groningen, The Netherlands

## Abstract

Lipid traits are known to be sex-differential, but the underlying molecular players are largely unknown. Herein, we aim to highlight differential molecular signatures in lipid metabolism between males and females by identifying proteins that are causally linked to lipids in a sex-specific or sex-differentiated manner. Since protein levels are downstream products of gene expression, proteomics data can be crucial to understand etiology of sex-differences in lipids metabolism.

We used sex-specific pQTL summary statistics for 2,923 circulating proteins measured in the UK Biobank Pharma Proteomics Project, along with sex-specific summary statistics of lipids from the Global Lipids Genetics Consortium. We combined these using two-sample Mendelian Randomization analyses and applied stringent multiple testing p-values correction and sensitivity analyses.

We identified several sex-specific significant causal links between protein levels and lipid phenotypes: 83 were exclusive to females and 82 to males, 46% of which would not have been identified using combined-sex GWASs. The estimated causal effect was instead significant in both sexes but substantially different in size for 39 causal relationships (p < 0.05). Intriguingly, several of these proteins were previously shown to be involved in inflammation and cardiometabolic diseases, such as Apolipoprotein(a) and lipoprotein lipase. Furthermore, two sex-specific proteins — LEPR and LPA — are molecular targets of emerging cardiometabolic therapies, highlighting the need for future investigations to determine whether these protein-level differences translate into sex-specific efficacy or safety in clinical trials and routine treatments.

The discovery of these sex-differences can provide important etiological insights into the management of lipid metabolism. Such knowledge may in turn improve the predictive use of sex-specific pQTLs and point to new therapeutic gender specific strategies to prevent cardiovascular disease.

**Supplementary Information:**

The online version contains supplementary material available at 10.1186/s13293-026-00934-5.

## Abstract (plain language summary)

Males and females often have different levels of lipids in their blood, such as cholesterol and triglycerides. These differences affect the risk of heart disease, but the biological reasons behind them are not fully understood. Proteins in the blood play an important role in how the body processes and transports lipids, and their levels can differ between sexes.

In this study, we investigated whether specific proteins influence lipid levels differently in males and females. To do this, we used genetic information from very large studies to examine nearly 3,000 blood proteins and their relationships with common lipid measures. Because genes are fixed at birth, this approach helps us understand which proteins are likely to play a causal role, rather than simply being correlated with lipid levels.

We found many protein–lipid relationships that were present only in females or only in males. We also identified several proteins that affected lipid levels in both sexes, but much more strongly in one than the other. Some of these proteins are already known to be involved in inflammation and heart-related diseases.

Importantly, two proteins that showed sex-specific effects are targets of new treatments for cardiometabolic diseases. This suggests that males and females might respond differently to certain therapies. Overall, our findings highlight the importance of considering sex differences when studying lipid biology and developing treatments to prevent cardiovascular disease.

**Supplementary Information:**

The online version contains supplementary material available at 10.1186/s13293-026-00934-5.

## Highlights

Lipid levels differ between females and males, but the molecular drivers of these differences remain poorly understood.

Using large-scale genetic and protein data, we identified proteins that influence blood lipid levels differently in females and males.

We found 83 protein–lipid relationships specific to females and 82 specific to males, as well as 39 shared relationships with markedly different effect sizes between sexes.

Several identified proteins are linked to inflammation and cardiometabolic disease, including apolipoprotein(a) and lipoprotein lipase.

Two sex-specific proteins, LEPR and LPA, are targets of emerging lipid-lowering therapies, underscoring the importance of considering sex differences in future drug development and clinical care.

**Supplementary Information:**

The online version contains supplementary material available at 10.1186/s13293-026-00934-5.

## Introduction

Cardiovascular diseases (CVD) are still the primary cause of death worldwide, with complex diagnosis and treatment further complicated by large sex differences in age of onset, symptoms and disease course [1–3]. Response to treatment can also vary drastically between males and females, mostly because many current therapies are based on clinical and pre-clinical research studies largely based on male subjects [2]. The existing knowledge gap of sex-specific or sex-differential biological mechanisms hampers the application of personalized medicine, leaving females underdiagnosed and undertreated.

One of the most important biomarkers of CVD is circulating lipid levels, for which epidemiological studies have highlighted substantial differences between sexes [4]. It is well known, for example, that pre-menopausal females have lower levels of low-density lipoprotein cholesterol (LDL) than same age males [5] however, after menopause, this pattern tends to reverse, with females having higher levels than males. In addition, high-density lipoprotein cholesterol (HDL) levels are higher among females of all ages compared to males [5]. Lipid levels also show a greater estimated heritability in females compared with males [6] especially for LDL and total cholesterol (TC) (> 1.3-fold difference), underling a possible genetic component behind this difference. Understanding the genetic determinants of sex difference in lipid levels and their molecular derivates could provide mechanistic insights into the biological processes underpinning CVD in both males and females and may improve CVD risk assessment and treatment.

Large scale genome-wide association studies conducted in millions of individuals, such as those carried out within the Global Lipid Genetic Consortium (GLGC) have highlighted the existence of genetic variants that are differentially associated with lipid levels between males and females [7]. However, their molecular derivates and if and how these impact lipid levels in each sex, remain poorly understood.

Herein, we aim to highlight differential molecular signatures in lipid metabolism between males and females by identifying proteins that are causally linked to lipids in a sex-specific or sex-differentiated manner. Since proteins are downstream products of gene expression and can be more directly related to biological processes, proteomics can be crucial to understand sex-differences in lipids metabolism and thus ultimately pinpoint to similar mechanisms in CVD.

To this end, we used publicly available sex‐stratified summary statistics of genetic determinants of protein and lipid levels, coupled with causal inference analyses. Specifically, we used sex-stratified cis-acting protein quantitative trait loci (pQTLs) summary statistics based on the UK Biobank Pharma Proteomics Project (UKB-PPP) [8] proteomic measurements to infer causal relationships between a total of 2,923 circulating proteins and five lipid levels using the sex stratified GWAS summary statistics from GLGC [7]. In addition, we used data from the Lifelines cohort to assess replication of our findings and to rule out the potential impact introduced by the known higher use of statins in males compared to females [9, 10].

We highlighted several proteins that exhibited significant sex-differentiated causal relationships with lipids, mostly involved in pathways related with CVD, insulin and inflammation. The discovery of these sex-differences provides important etiological insights into the regulation of lipid levels. Such knowledge may, in turn, demonstrate the importance of sex-specific pQTLs and point to new or revised therapeutic sex specific strategies to prevent CVD.

## Materials and methods

### Populations studied

In this study we used the sex-specific cis-pQTL GWAS summary statistics of 2,923 circulating proteins measured in the UKB-PPP, a precompetitive consortium of 13 biopharmaceutical companies [11], as exposure for our causal inference analyses [8]. The UK Biobank proteomic measurements were conducted by antibody-based Olink technology Explore 1536 platform which uses Proximity Extension Assay [12]. Further details about antibody-based proteomic measurements and quality control have been described elsewhere [11]. Further details about the sex-stratified proteomic GWASs have also been described elsewhere [8]. Briefly, the analysis was restricted to individuals of European ancestry from the UK Biobank for whom both genotype and proteomics data were available. Specifically, ancestry outliers and samples failing genomic or proteomic quality control were excluded, resulting in a final sample of 48,017 individuals (25,904 females [53.9%] and 22,113 males [46.1%], aged 49–60). Protein abundances were inverse rank normalized and adjusted for technical covariates (e.g., blood draw timing, fasting status). Sex-stratified proteomic GWASs were then conducted using these residuals with REGENIE v.3.4.1 software, including age, age^2^, and proteomic batch as covariates [8]. Only individuals with matching sex chromosome and recorded/self-reported sex were included to avoid potential genotyping issues [8].

We also used sex-specific GWAS summary statistics of HDL, non-high-density lipoprotein cholesterol (non-HDL), LDL, total cholesterol (TC) and TG, downloaded from the GLGC (https://csg.sph.umich.edu/willer/public/glgc-lipids2021/results/sex_and_ancestry_specific_summary_stats/), [7] as outcomes. Sample size for these five traits varied between 459,578 and 581,618 for females and between 401,908 and 713,344 for males. In addition, we used the Lifelines cohort, a large biobank from Northern Netherlands [9, 13], to replicate the significant findings using the same pipeline as implemented in the GLGC to derive GWAS statistics for lipids. Furthermore, we used this cohort to evaluate the impact of statins (lipid-lowering medications) and derived additional GWAS statistics after excluding individuals under such medications.

### GWAS of lipid levels in the Lifelines cohort

Lifelines Cohort Study data was used for replication of our main results and to evaluate the potential impact of statin usage in the results. Lifelines Cohort Study is a multidisciplinary prospective population-based cohort study that utilizes a unique three-generation design to investigate health and health-related behaviors in 167,729 individuals from the northern Netherlands. Lifelines employs a broad range of investigative procedures to assess the biomedical, socio-demographic, behavioral, physical, and psychological factors that contribute to health and disease, with a special focus on multi-morbidity and complex genetics [9, 13].

Overall, 64,197 Lifelines individuals had both phenotype and genotype data available. Genotype data of Lifelines participants were generated in two batches. Batch one (UGLI1) was genotyped on the Infinium Global Screening Array MultiEthnic Diseases version 1.0 and imputed using HRC v 1.1 as the reference. 35 individuals were predicted to be of non-European descent based on PCA. Detailed data processing description can be found in Lopera-Maya et al. [14]. The second batch of samples (UGLI2) was genotyped on the FinnGen Thermo Fisher Axiom® custom array and imputed using HRC v 1.1 as the reference. 88 individuals were predicted to be of non-European descent based on PCA. These two genotype batches were merged and filtered keeping genetic variants with minor allele frequency (MAF) > 0.05, call rate > 0.95, Hardy–Weinberg equilibrium p-value > 1e-6. Detailed genotype quality control description for both batches is available on the Lifelines wiki page: https://wiki.lifelines.nl/doku.php?id=ugli. We conducted sex-stratified GWAS in 2 groups: all individuals, regardless of the medication use, and individuals who did not report statin usage (as determined by ATC code C10AA) (N = 60,875). Percentage of females was 60% and 61% in the two groups respectively.

Lipid levels data were processed in accordance with Graham et al. [7]: 1) we log-transformed triglyceride levels; 2) we adjusted each lipid in each phenotype group and each sex for covariates by regressing out age, age squared and taking the residuals of the linear model; 3) we inverse-rank transformed these residuals. For the analysis of all individuals together irrespective of their medication status, we adjusted the lipid levels of individuals on statins to estimate their pre-medication levels by dividing the LDL cholesterol value by 0.7 and the total cholesterol value by 0.8.

GWAS was done using REGENIE v3.4.1 [15]: the first step (whole genome regression) was run on a subset of SNPs genotyped on the GSA array, with parameters –bsize 200 –loocv; the second step was run on all imputed filtered SNPs using –bsize 200 parameter. Genotyping batch was used as covariate in both steps.

### Mendelian randomization analyses

We performed linkage disequilibrium (LD) clumping using a 10 Mb window and an r^2^ cutoff ≤ 0.001 for all the proteins-related GWAS available with UKB-PPP proteomic measurements, to select common and genome-wide significant independent hits; this step was carried out with PLINK (version 2.0) [16].

The variants selected by PLINK were then used as instrument variables (IVs) to perform two-sample Mendelian randomization (MR) analyses [17]. MR was carried out using only in cis-acting variants, including variants located on chromosome X. Cis pQTLs were defined as genetic variants located within 500 kb of the gene encoding the protein. We performed the main MR analyses using the combined (all samples) as well as the sex-stratified pQTL GWAS summary statistics from the UKB-PPP as exposures [8] and the combined as well as the sex-stratified GWAS summary statistics of HDL, non-HDL, LDL, TC and TG from the GLGC [7] as outcomes. When IVs were absent in the outcome dataset, we utilized the LDproxy function in R [18] to identify suitable proxies in linkage disequilibrium ( r^2^ > 0.8).

We performed two-sample MR using the standard inverse-variance weighted (IVW) regression—or the Wald ratio method in case of a single instrument variable—as main methods to declare significance. In addition, when multiple IVs were available, we used MR-PRESSO [19] to minimize the risk of horizontal pleiotropy, and the weighted median-based method. We specifically tested for the presence of horizontal pleiotropy in all IVs by calculating intercept values from the MR Egger regression [20]. Furthermore, we visually inspected scatter plots across all MR methods tested in our study and performed leave-one-out sensitivity analyses to identify if a single SNP was driving an association. We performed the two-sample MR analyses with the R package TwoSampleMR, version 0.6.8 [17].

We defined a causal relationship significant only if the following conditions were met: 1) p-value ≤ 0.05 after false discovery rate (FDR) correction (Benjamini–Hochberg method) [21] in IVW or Wald ratio, 2) nominal p-value < 0.05 for sensitivity MR methods MR-PRESSO and Weighted Median, and 3) MR-Egger intercept p > 0.05 and 4) the association was not driven by a single SNP as determined by leave-one-out analysis and this SNP had not been removed by MR-PRESSO.

Since for MR conducted on one single IV, sensitivity analyses cannot be performed, we further screened the Wald ratio results (the results of MR conducted on single IVs) and kept only proteins explaining at least 1% of the total variance in protein levels. Among the significant causal relationships detected, we defined “sex-specific” those relationships that met the significance criteria above (all four conditions) in one sex and the unadjusted p-value in main methods (IVW or Wald ratio) was ≥ 0.05 in the other sex.

As additional sensitivity analysis for the selected causal associations, we performed reverse MR to confirm the lack of significant reverse causation or confounding. To this end, we used the sex-stratified GWAS of lipids as exposure and sex-stratified GWAS of protein levels as outcome and selected IVs applying the same pruning criteria described above.

Since many protein-lipid relationships may be significant in one sex but still show nominal significance in the other sex or be associated with both sexes in a differentiated manner, we systematically evaluated differences in causal estimates detected in males and females. Specifically, we applied the Cochran’s Q test [22] to test for significant differences in the causal estimates from IVW (or Wald ratio for single variants) and selected those showing Cochran’s Q p-value < 0.05. To account for correlations among proteins and potential horizontal pleiotropy, we performed multivariable Mendelian randomization (MVMR) following the univariable MR analysis, using the MVMR R package [42]. MVMR analyses were conducted separately in males and females. For each sex, we selected proteins that showed statistically significant associations in the primary univariable MR analyses and jointly modelled them as exposures in a multivariable framework. We combined all genetic instruments of each protein selected for univariable MR and derived the effect size and standard error for all proteins. These SNPs were then subjected to a second step linkage disequilibrium (LD) clumping (10,000 kb window, r^2^ < 0.001) considering the minimum p-value across proteins as reference p-value and the same European reference panel used for LD clumping in the univariable MR. SNPs alleles and effect of all exposure and lipid outcomes were aligned. Multivariable MR estimates were obtained using an inverse-variance weighted (IVW) approach (function *ivw_mvmr* in MVMR R package). To assess instrument strength, we calculated conditional F-statistic for each exposure. Horizontal pleiotropy was evaluated using the multivariable Q-statistic.

Finally, we performed replication of significant findings and evaluation of the influence of statin therapy by repeating the MR analyses using the same exposures (proteins), and as outcomes sex-stratified GWAS summary statistics of HDL, non-HDL, LDL, TC and TG derived from the Lifelines cohort. We used the following criteria to define replication: IVW, Wald ratio, Weighted median, MR-PRESSO p-value ≤ 0.05, all leave-one-out p-value < 0.05 and MR Egger intercept p-value > 0.05 in the same sex-specific analysis as in the main analysis, and same direction of causal effect.

**Fig. 1:**
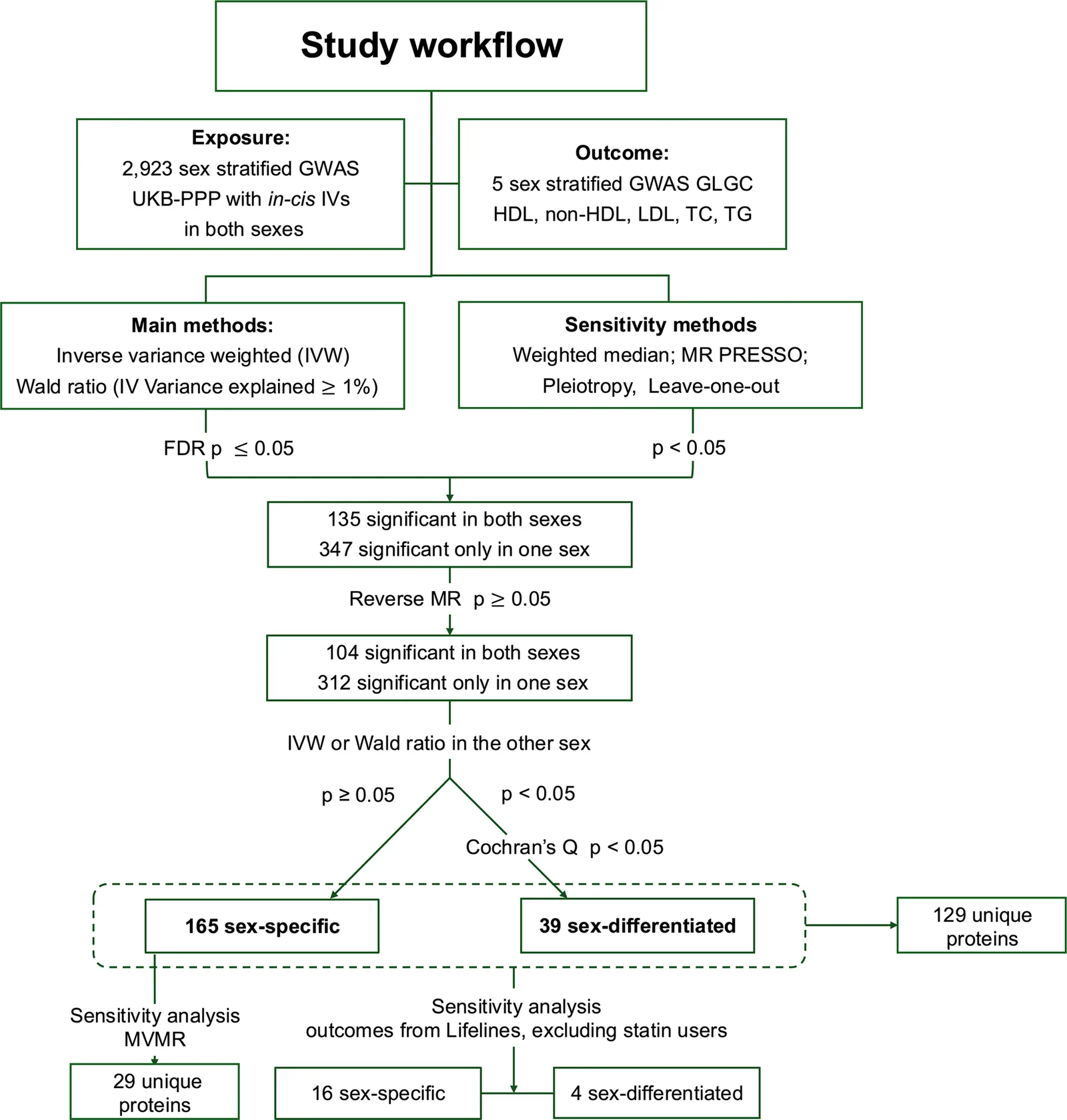
Flow chart of the different data sources and methods used in this study. The figure provides a schematic representation of the methods, filtering steps and results obtained. GWAS, genome-wide association studies; UKB-PPP, UK Biobank Pharma Proteomic Project; HDL, high-density lipoprotein cholesterol; non-HDL, non-high-density lipoprotein cholesterol; LDL, low-density lipoprotein cholesterol; TC, total cholesterol; TG, triglyceride; GLGC, Global Lipid Genetic Consortium; IVs, instrument variables; MVMR, Multivariable Mendelian Randomization

A flow chart of the different data sources and methods used in this study is shown in Fig. 1.

### Enrichment analyses

To understand the biological mechanisms and pathways highlighted by proteins showing sex-specific and sex-differentiated causal relationship with lipids, we performed the Search Tool for the Retrieval of Interacting Genes/Proteins (STRING) analysis (https://string-db.org). For the enrichment analysis we defined the background all the 1,470 proteins tested in our MR analyses. We investigated the gene ontology (GO) pathways within these clusters using a combination of the lowest FDR with a strength value > 0.50. Strength in GO reflects the level of confidence or support for the functional relationship between different genes or terms based on available biological evidence.

### Functional mapping with FUMA

Functional annotation of genes of interest was performed using the GENE2FUNC module of FUMA (Functional Mapping and Annotation of GWAS) [23]. The input gene set consisted of genes encoding the sex-specific proteins identified in our study, while the background gene set comprised all genes encoding proteins analyzed in our study. We performed enrichment analyses within differentially expressed genes pre-calculated in the GTEx v8 RNA-seq data across 54 specific tissue types and 30 general tissue categories. Differential expression was assessed by FUMA using two-sided t-tests with Benjamini–Hochberg false discovery rate (FDR) correction as implemented in the platform. All analyses were conducted using default FUMA settings unless otherwise specified.

### Clinical relevance annotation

Clinical relevance of the identified proteins was assessed by integrating information from gene function databases, genetic association resources, and drug annotation platforms. Gene symbols and full gene names were retrieved from Ensembl BioMart (Ensembl Release 115, accessed 5 December 2025), and functional summaries were obtained from NCBI Gene using the Entrez Gene Summary field (accessed 5 December 2025). Genetic associations were annotated using the NHGRI–EBI GWAS Catalog full associations file (release e115_r2025-11–12, downloaded 12 November 2025). Gene coordinates from BioMart were expanded by ± 10 kb and overlaps with variants annotated in the GWAS Catalog were identified using genomic range intersections. Drug and therapeutic annotations were obtained from the Open Targets “known_drug” dataset (accessed 5 December 2025) and matched to genes via Ensembl gene identifiers.

## Results

### Mendelian randomization analyses

A total of 1,882 and 1,910 proteins were associated with at least one cis genomic variant in males and females, respectively, in the UKB-PPP dataset and were therefore selected as exposures for two-sample MR analyses. MR was carried out using all potential IVs acting only in cis (Supplementary Table 1). To describe results along the text, we will use the name of the gene encoding the assay, instead of using protein names or protein identification numbers (UniProtID) (for example, we will use MMP7 instead of P09237).

After removing causal relationships driven by just one single IV (Supplementary Table 2), we identified a total of 347 non-pleiotropic (p-value ≤ 0.05 in MR Egger pleiotropy test) formally significant causal relationships in only one sex (FDR ≤ 0.05 in IVW and Wald ratio, nominal p-value in weighted median and MR-PRESSO), while in the other sex the evidence of an effect was null or not conclusive (Supplementary Table 3). We further removed relationships showing evidence of reverse causality (Supplementary Table 4) and kept a total of 312 non pleiotropic links; among them, 165 met our strict criteria for being defined sex-specific (IVW p-value or Wald ratio ≥ 0.05 in the other sex) (82 male-only and 83 female-only) (Supplementary Table 5 and Fig. 1).

Scatter plots and leave-one-out plots performed across all MR methods tested in our study are available at https://github.com/Sanna-s-LAB/Sex_dimorphism_MR_Proteomics/tree/main/Supplementary_material.

**Fig. 2:**
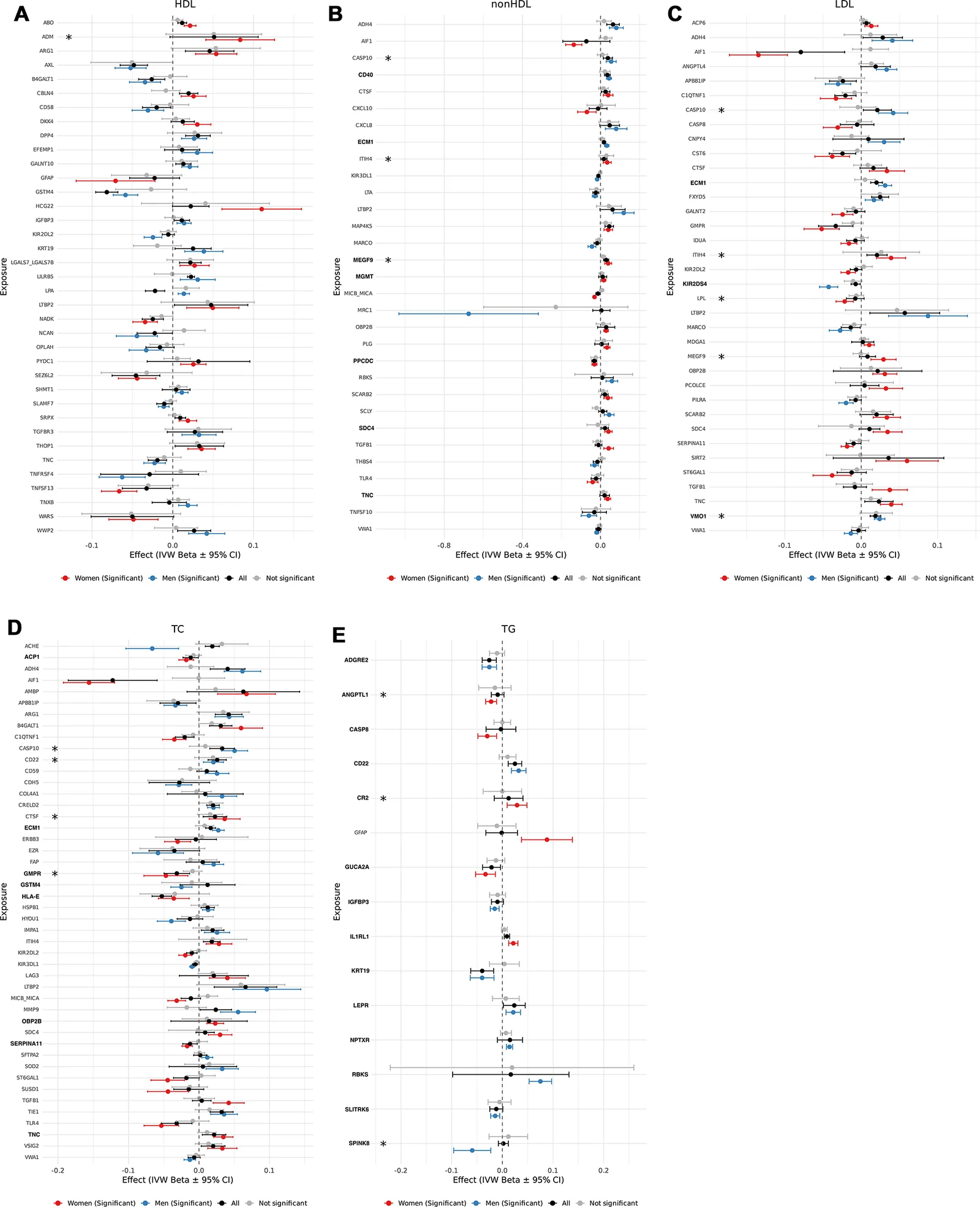
Forest plot of the proteins showing sex-specific causal effects. The figure provides a graphical representation of causal estimates according to IVW method and corresponding confidence intervals of protein effect on lipid levels for all the significant causal links detected. Proteins showing a significant association after multivariable MR are indicated in bold. Significance was determined using two-tailed p-values from the IVW method, with adjustment for multiple testing. **A**, HDL, high-density lipoprotein cholesterol; **B**, non-HDL, non-high-density lipoprotein cholesterol; **C**, LDL, low-density lipoprotein cholesterol; **D**, TC, total cholesterol; **E**, TG, triglycerides.* Indicates significant replication in the Lifelines cohort excluding individuals under lower lipid medication treatment (statins). IVW, inverse variance weighted method

**Fig. 3:**
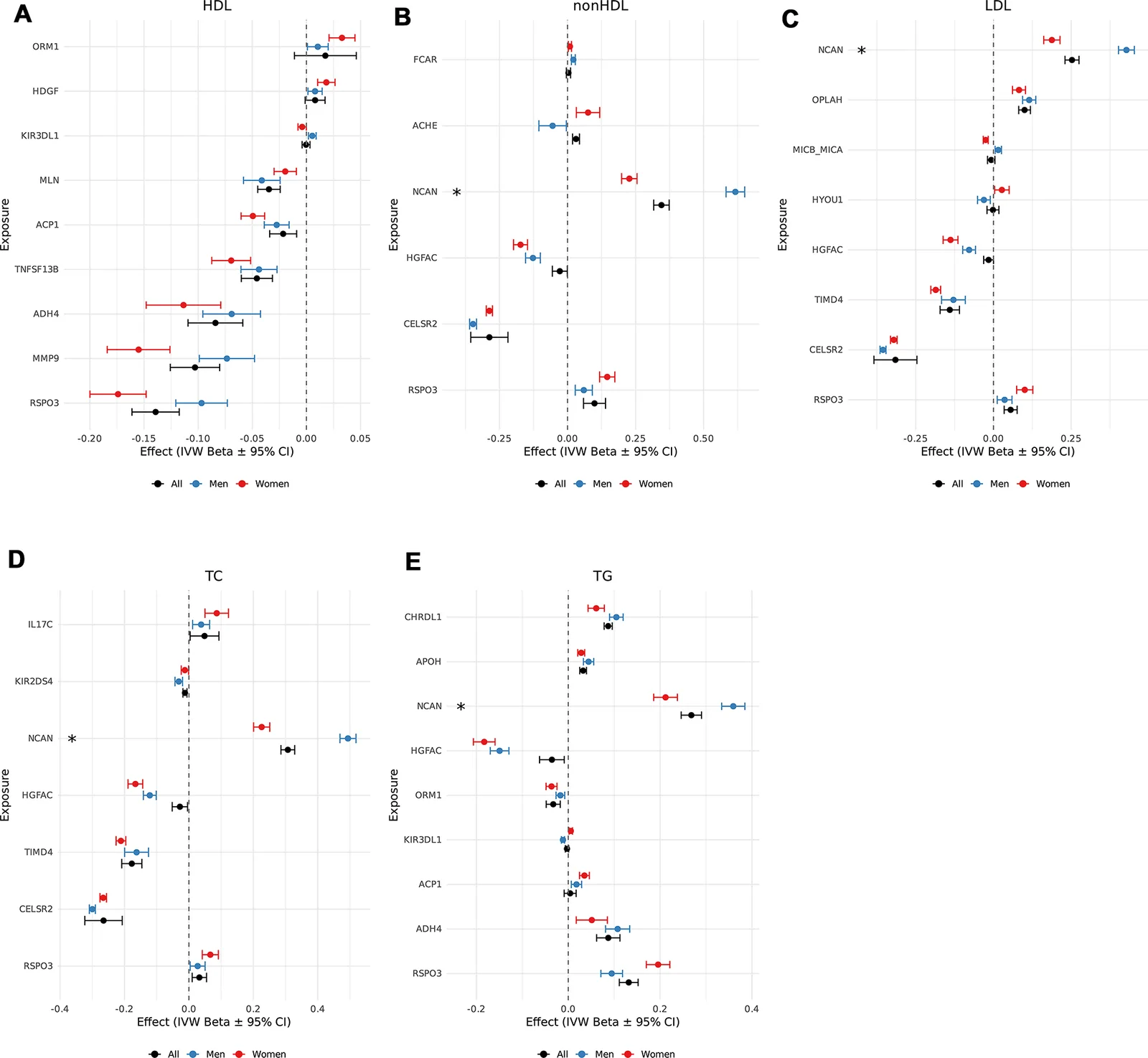
Forest plot of the proteins showing sex differentiated effects. The figure provides a graphical representation of causal estimates according to IVW method (two-tailed p-value) and corresponding confidence intervals of protein effect on lipid levels for all the proteins showing significant differences in causal estimate detected by Cochran’s Q test. **A**, HDL, high-density lipoprotein cholesterol; **B**, non-HDL, non-high-density lipoprotein cholesterol; **C**, LDL, low-density lipoprotein cholesterol; **D**, TC, total cholesterol; **E**, TG, triglyceride.* Indicates significant replication in the Lifelines cohort excluding individuals under lower lipid medication treatment (statins). IVW, inverse variance weighted method

We evaluated effect size differences between males and females for the 147 causal relationships formally significant only in one sex but not acting as sex-specific (312–165), and additional 104 relationships that were formally significant in both sexes. We used the Cochran’s Q test on the estimated causal effects from the main MR methods: IVW and Wald ratio. We identified 39 relationships with significant differences in causal estimates (Cochran’s Q p < 0.05) (Supplementary Table 5). For example, the three proteins with the most pronounced sex differentiated causal estimates were: Neurocan core protein (NCAN), on LDL, non-HDL, TC, and TG; Cadherin EGF LAG seven-pass G-type receptor 2 (CELSR2) on non-HDL; and Killer cell immunoglobulin-like receptor 3DL1 (KIR3DL1) on TG (full results in Supplementary Table 5). With the main aim of investigating differences in causal relationships between sexes, in Fig. 2 we highlighted the 165 (83 females-only, 82 males-only) significant sex-specific protein-lipid links detected by the MR analyses and in Fig. 3 we showed the 39 significant relationships detected by the Cochran’s Q test. Overall, we observed that the direction of the causal estimates was generally consistent between females and males, and the sex differences laid in the magnitude. However, for a total of 41 relationships, we detected opposite estimate between the sexes (see “yes” in the “opposite_estimate” column in Supplementary Table 5)— for example, the protein NCAN showed a negative causal estimate for HDL in males and a positive estimate in females, whereas Allograft Inflammatory Factor 1 (AIF1) showed negative estimates for non-HDL and LDL in females and positive causal estimates in males. To account for potential bias arising from correlated protein exposures and shared biological pathways, we performed MVMR as an additional analysis. Analyses were conducted separately in males and females, restricting the models to proteins that were classified as sex-specific in the primary univariable MR analyses. Within each sex, these proteins were jointly included as exposures in a multivariable framework, enabling estimation of their independent effects on lipid traits while accounting for correlation between proteins and potential horizontal pleiotropy. Across sexes, MVMR identified a total of 32 significant, non-pleotropic and with an F-statistic > 10 associations, including 16 in women and 16 in men, involving 29 sex-specific proteins. Only two proteins showed association with multiple outcomes: TNC was associated with non-HDL cholesterol and total cholesterol in women, while ECM1 was associated with LDL cholesterol, non-HDL cholesterol, and total cholesterol in men (Supplementary Fig. 4B). These results suggest that most identified protein–lipid associations are outcome-specific after accounting for correlation among protein exposures (Supplementary Table 6). For certain outcomes—notably HDL in both sexes and LDL in females— we detected evidence of heterogeneity among instruments (Q-statistic p-value < 0.05) consistent with potential horizontal pleiotropy (i.e., genetic variants influencing the outcome via pathways not captured by the included exposures). These findings suggest that the associated instrumental variables may not be fully valid within the MVMR model, and thus results should be interpreted with caution. Enrichment analyses of sex-differentiated and sex-specific proteins identified through univariable MR and MVMR did not yield significant results.

Among the identified proteins in the univariable MR, we noted known players in lipid metabolism like the lipoprotein lipase (LPL) associated with increased levels of LDL only in women or the Apolipoprotein(a) (LPA) associated with increased levels of HDL only in males. In addition, we detected novel important relationships with lipids, like the Leptin Receptor (LEPR), a key player in the regulation of energy balance and body weight control [24] and the Matrix metalloproteinase-9 (MMP9), implicated in atherosclerotic plaque rupture [25], showing in our study an higher causal effect on TG and on TC, respectively only in males.

### Replication in the Lifelines cohort

We performed a GWAS of lipid levels in the Lifelines Study Cohort to replicate our MR results and to check if usage of statins (lipid-lowering medications) could have introduced bias in our causal estimates. In this cohort, we tested for replication the 165 sex-specific and the 39 sex-differentiated relationships. We declared replication of sex-specific when the causal relationship was considered sex-specific according to our definition in the same sex-group and with the same direction as in the main analysis. We declared replication of sex-differentiated when the Cochran’s Q test p was < 0.05 and the direction of single causal estimates were the same as in the main analysis.

When using the sex-specific GWASs derived in the full Lifelines Study cohort (N ≃ 38,554 in females and N ≃ 25,647 in males) we successfully replicated a total of 20 relationships, 13 in females and 7 in males. All the 20 sex-specific relationships detected except 4 also replicated when using the GWAS derived excluding statin users (N ≃ 36,982 in females and N ≃ 23,895) (see “yes” in the column Consistency_GLGC_Lifelines_sexspecific in Supplementary Table 6). The four relationships that did not show replication after removing statin users involve the following three proteins: Syndecan-4 (SDC4), FXYD domain-containing ion transport regulator 5 (FXYD5), and Cathepsin F (CTSF). A direct link between these three proteins and statins was not reported previously. A total of 4 sex-differentiated relationships replicated in the Lifelines cohort in the full dataset and excluding individuals using statins (see “yes” in the column Consistency_QCochran_Lifelines_sexspecific in Supplementary Table 7).

### Characterization of proteins selected

We identified a total of 129 unique proteins from the previous analyses, specifically 117 acting sex-specific and 22 in a sex-differentiated manner; 10 of them behaved as sex-specific or sex-differentiated depending on the outcome. A full list of the 129 significant proteins together with their function is shown in Supplementary Table 8. Several of these proteins are directly linked to lipid metabolism, such as LPL [26], LPA [27], and LEPR [28]. Others, including C-X-C motif chemokine ligand 8 (CXCL8) [29], are associated with inflammation—a key contributor to metabolic syndrome. Additional notable proteins include insulin-like growth factor binding protein 3 (IGFBP3), which has been linked to T2D [30], and MMP9, which is implicated in atherosclerosis [31].

**Fig. 4:**
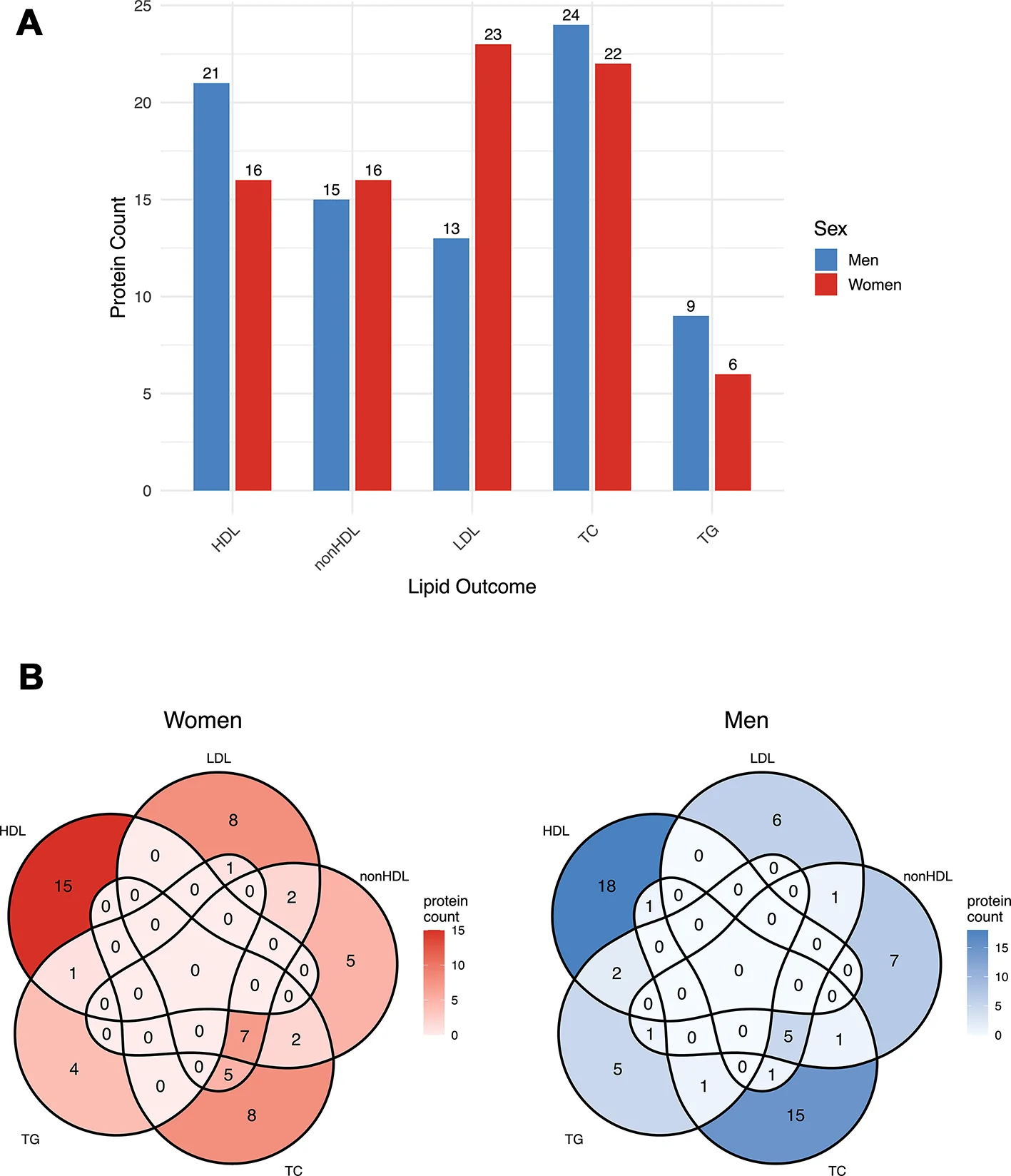
Sex-specific impact of causal relationships. **A**. The barplot provides a count of proteins selected in each outcome divided by sex. **B**. Venn diagrams showing the number of proteins shared or specific to lipid traits in males and females. For this plot in both panels, only sex-specific relationships are considered. HDL, high-density lipoprotein cholesterol; non-HDL, non-high-density lipoprotein cholesterol; LDL, low-density lipoprotein cholesterol; TC, total cholesterol; TG, triglyceride

Of note, in all the outcomes, except non-HDL and LDL, males showed the highest number of sex-specific proteins detected (Fig. 4A). Moreover, most proteins within each sex were outcome-specific and not shared across the five lipid-related outcomes analyzed (Fig. 4B). In females, for example, 40 proteins were uniquely associated with a single lipid trait, with the highest number (15 proteins) linked exclusively to HDL, while only 18 proteins were associated with more than one lipid trait.

Notably, none of the sex-specific proteins was causally linked with all five lipid traits together, in either sex. When outcomes were not considered, only 5 out of the 117 proteins were causally linked to both females and males, underscoring that the sex-specific relationships detected exist not only for the exposure–outcome pairs but also for individual proteins.

### Functional mapping with FUMA

We performed tissue enrichment analysis using FUMA to identify tissues in which the genes mapped by the sex-specific proteins are preferentially expressed. Sex-specific proteins were significantly enriched in the genes up-regulated in breast among all specific and general tissues (Supplementary Fig. 5), (p-value after Benjamini–Hochberg FDR correction = 0.040 and 0.022 respectively).

### Clinical relevance annotation

When assessing the pharmacological relevance of the sex-specific and sex-differentiated protein-encoding genes identified in our study, we found that a total of 30 genes are molecular targets of drugs that are approved or under investigation. All annotations detected were integrated into the Supplementary Table 8 summarizing gene function, evidence of genetic associations, and known drug targets.

## Discussion

We studied the sex-specific causal relationships between 2,923 circulating proteins measured in the UKB-PPP, and lipid metabolism, specifically with HDL, non-HDL, LDL, TC and TG using GWASs from the Global Lipids Genetics Consortium. From this study, several key findings emerge. First, we identified a total of 83 and 82 robust causal protein-lipids links that were specific to females and males, respectively. Notably, nearly half of these associations (46%; 76 protein–lipid associations) would not have been detected using combined-sex GWASs. This reinforces the importance of conducting sex-specific GWASs at both phenotypic and molecular levels.

Second, we detected a total of 39 protein–lipids links that showed sex-differentiated causal effects, highlighting their potential involvement in sex specific biological pathways.

Overall, these links involve 129 unique proteins, including 117 with sex-specific effect, 22 acting in a sex-differentiated manner, and 10 displaying either sex-specific or sex-differentiated-effects depending on the outcome considered.

Finally, after accounting for correlation among proteins using MVMR, we identified 29 proteins representing robust, statistically independent signals within each sex-stratified analysis.

It is noteworthy that most of the sex-specific associations identified in the univariable MR analyses were not retained in the MVMR models. Several factors may contribute to this observation. First, underlying pleiotropy among protein exposures for certain outcomes is likely, as reflected by low conditional F-statistics for some proteins and significant Q-statistics in the MVMR analyses. Second, optimal application of MVMR would require either exposure GWASs derived from independent cohorts or estimation of exposure covariance using individual-level data, neither of which was feasible in the present study. Third, the large number of exposures included is expected to reduce statistical power.

Most sex-specific proteins within each sex were outcome-specific and not shared across the five lipid-related outcomes analyzed. We also observed a higher proportion of sex-specific relationships in males for all traits (except for LDL and non-HDL), suggesting that, in females, regulation of lipids is more strongly influenced by non-genetic factors, for example sex hormones fluctuations during life stage. Of note, the difference in discovery is unlikely to be due to differences in statistical power, since the sample size of females-only GWASs was overall similar than the males-GWAS for both proteins and lipid GWASs. It is worth noting that, while circulating protein levels frequently differ between sexes, current evidence suggests that their genetic determinants are largely shared [8], implying that the sex-specific causal effects observed here are more likely driven by downstream biological context rather than sex-dependent genetic regulation of protein expression.

### Comparison with prior literature

Among the proteins identified in our study, some are involved in various physiological processes, and their levels or activity are differently expressed in males versus females. MMP9 for example, emerged as a key candidate, being selected as sex-specific for TC. Specifically, we highlighted a causal relationship between increased levels of this protein and increased levels of TC in males (Fig. 2D). A previous study highlighted that male rat aortic smooth muscle cells exhibit significantly higher MMP9 gene expression, protein levels, and enzymatic activity compared to female [32]. However, to our knowledge no published evidence exist on the opposite causal effect of MMP9 on TC in males and females.

Another protein worth mentioning is the Angiopoietin-like 4 (ANGPTL4), a multifunctional glycoprotein that plays key roles in lipid metabolism, vascular biology, and inflammation [33]. It has been previously seen in mice that ANGPTL4 expression is higher in males than females in both liver and adipose tissue, and this difference is functionally relevant—male mice show a greater shift in plasma triglycerides and cholesterol when ANGPTL4 is genetically altered [34]. In line with this, our study highlighted a causal link of increased levels of ANGPTL4 on increased levels of LDL only in males (Fig. 2C).

Lipid levels have recently emerged as independent factors contributing to the onset of heart failure with preserved ejection fraction [35]. A previous study highlighted that in heart failure with preserved ejection fraction, females exhibited higher circulating LPL and IGFBP‑3 levels than males [36]. In our study we detected a causal relationship of increased IGFBP-3 levels with increased level of HDL (Fig. 2A) and decreased levels of TG in males only, replicated also with the MVMR analyses (Fig. 2E) and a causal sex-specific link of LPL and lower LDL in females which replicate also in the Lifelines cohort (Fig. 2C). Other previous evidence suggested that LPL activity shows a clear sex difference with fundamental differences in the regulation of triglyceride uptake between males and females in adipose regions. Females show stronger associations between adipocyte size and LPL activity in specific fat depots, whereas males show different regional patterns [37]. Therefore, our results suggest that a differential molecular effect of these proteins could be involved in sex dimorphism of heart failure with preserved ejection fraction.

In addition, we found evidence of sex-specific causal relationship of the Toll-Like Receptor 4 (TLR4) and decreased levels of TC (Fig. 2D) and non-HDL (Fig. 2B) only in females. Emerging evidence indicates that TLR4 signaling is modulated by sex hormones and exhibits sex-specific regulatory patterns with implications for cardiovascular aging and immune function [38]. Toll-like receptors (TLRs) comprise a family of pattern recognition receptors that, upon activation, initiate pro-inflammatory signaling cascades. These receptors are broadly expressed within the cardiovascular system, where accumulating data implicates them in the sexual dimorphism observed in cardiovascular pathophysiology. Notably, sex-specific differences in TLR activity persist with advancing age, suggesting a potential paradigm shift in understanding the mechanisms that underlie sex differences in cardiovascular aging [38].

### Biological and clinical relevance

We observed that genes encoding the sex-specific proteins are up-regulated in breast tissue. This finding aligns with known biological sex differences in breast morphology, endocrine regulation, and disease susceptibility. This observation is consistent with previous evidence that many genes in breast tissue exhibit sex-biased expression and that the number of differentially expressed genes between sexes is by far largest in breast compared to most other tissue [39]. Of note, receptors for estrogens, androgens, and other sex steroids are expressed in hormonally responsive tissues including breast [40]. We thus speculate that the tissue enrichment in breast observed in our analysis may reflect a hormone receptor–mediated regulatory hub relevant to metabolic and lipid associated pathways across sexes.

When assessing the pharmacological relevance of the sex-specific and sex-differentiated protein-encoding genes identified in our study (Supplementary Table 8), we found that a total of 30 genes are molecular targets of drugs that are approved or under investigation. For example, for two proteins, LEPR (leptin receptor) and LPA (lipoprotein(a)), there are drugs under investigation at phase III which are specifically tailored as cardiometabolic therapies. This observation has potential clinical significance: sex-specific regulation of LEPR or LPA levels could influence individual responses to leptin- or Lp(a)-targeted treatments or uncover sex-dependent pathogenic mechanisms in metabolic and cardiovascular disease. In particular, the association between LPA and Lp(a)-lowering therapies (e.g., Pelacarsen, Olpasiran) suggests that sex-specific differences in circulating Lp(a)-related proteins may have implications for cardiovascular risk stratification and therapeutic targeting. Similarly, LEPR — targeted by Metreleptin or Mibavademab — may reflect sex-specific modulation of leptin signaling and metabolic homeostasis. Collectively, these findings provide a rationale not only for mechanistic follow-up studies investigating how sex modifies LPA or LEPR expression and function, but also for exploring whether sex-specific protein differences translate into differential drug responses, efficacy or disease risk profiles.

Beyond LPA and LEPR, the druggable evidence for the additional 28 genes highlights the need to investigate sex-specific efficacy and response on the respective target diseases and also encourages studies to test potential repurposing of these drugs for lipid related therapies in a sex-specific manner.

### Strengths and limitations

Our study is the largest and most comprehensive study of sex-specific causal associations of protein biomarkers with lipid metabolism to date. Other strengths of our study include the large sample size, the most recent and powerful GWAS summary statistics used as exposures and outcomes, the range of sensitivity analyses increasing robustness of our findings and the replication of the results in a different cohort. We applied robust methods with special assumptions about the behavior of pleiotropic variants, such as Egger intercept [41], which assumes pleiotropic effects are uncorrelated with the genetic associations with the risk factor, and the MR-PRESSO [19], which excludes outlier variants as being potentially pleiotropic. In addition, we performed reverse MR to exclude any possible bias due to reverse causation or confounding, thereby strengthening the validity of our results.

We also acknowledge the study limitations. First, we were limited to using pQTLs currently available in the UKB-PPP, and thus missed proteins not measured in this cohort. Second, the participants included in the genetic analyses were of European ancestry and the age range of UKB-PPP participants is very narrow (49–60 years old). Hence, our results may not be generalizable to other ethnic groups with significantly different ages and different lipid metabolism profiles. In addition, our analyses relied exclusively on summary-level data, as we did not have access to the relevant individual-level data for the proteomic measurements used, precluding one-sample MR for further validation. Finally, we performed replication using a different data set as outcome: the Lifelines cohort (with and without individuals under lower-lipid medication treatment). While this provides already some evidence of results being genuine, a formal replication would require to also change the exposure cohort (UKB-PPP).

## Conclusions

Our work highlighted several proteins with sex-specific and sex-differentiated causal impact on lipid levels, results that could be obtained only with the availability of sex-specific GWASs. Some of these proteins were also drug targets for cardiometabolic diseases, while others were targets for other chronic diseases. The discovery of these sex-differences can thus provide important etiological insights into the management of lipid metabolism. Such knowledge may in turn improve the predictive use of sex-specific pQTLs and point to new therapeutic gender specific strategies to prevent CVD. Finally, we enhance the need to perform sex-stratified pQTL mapping in all future GWAS of any complex trait and disease as route to derive relevant insights into potential genetic drivers of sex dimorphism not only in lipid metabolism.

## Supplementary Information

Supplementary Material 1

Supplementary Material 2

## Data Availability

Sex-stratified GWAS summary statistics for all proteomic measurements available through UKBB-PPP are available at omicscience.org. Further information can be found at Koprulu et al. (2025) [8]. GWASs data from the GLGC consortium were downloaded from https://csg.sph.umich.edu/willer/public/glgc-lipids2021/results/sex_and_ancestry_specific_summary_stats/. The scripts used for all the analyses to reproduce the main results, as well as additional figures are publicly available in the GitHub repository: https://github.com/Sanna-s-LAB/Sex_dimorphism_MR_Proteomics. Conversely, the Lifelines Cohort Study data that was used for replication is not available for direct download and can be requested, for a fee, by filling the application form at https://www.lifelines.nl/researcher/how-to-apply/apply-here. Lifelines will not charge an access fee for controlled access to the full dataset used in the manuscript, for the specific purpose of replication of the results presented in this Article or for further assessment by the reviewers, for a period of three months. Researchers interested in such a replication study or review assessment can contact Lifelines at research@lifelines.nl.
