# Supplementary material for: Exploring sex-specific causal links between thousands of proteins and lipid metabolism using the UK Biobank Pharma Proteomics Project data
Source: Biol Sex Differ. 2026 Jun 16;17:148. doi: 10.1186/s13293-026-00934-5 (PMC13505004; doi:10.1186/s13293-026-00934-5)
Supplement: Supplementary file 1 [file 13293_2026_934_MOESM2_ESM.docx]

**Supplemental Material**

**Supplementary Tables**

**Supplementary Table 1.** Mendelian randomization results for all proteins in females, in males and in the full dataset.

The table reports the results for all proteins analyzed in the main MR analysis. Two-tailed p-values are reported for all methods analyzed, including IWW, Wald ratio, MR Egger, MR-PRESSO, and Weighted median. The column SignificantBysex highlighted the significant results based on sex: 1, significant results in females; 2, significant results in males; 3, significant results in both sexes; 4, significant results showing a variance explained < 0.01 in one of the two sexes; 5, no significant results.

Abbreviations: HDL, high-density lipoprotein cholesterol; non-HDL, non-high-density lipoprotein cholesterol; LDL, low-density lipoprotein cholesterol; TC, total cholesterol; TG, triglyceride; nsnp, number of single nucleotide polymorphisms; b, causal effect estimate; se, standard error, pval, two-tailed p-value, ple, pleiotropy; fdr, false discovery rate; het, heterozygosity; IV, instrument variable; exp_var, variance explained.

**Supplementary Table 2.** Leave-one-out results of the significant causal relationships.

The table lists leave-one-out results for all protein-lipid relationships showing FDR <0.05 and two-tailed p-value >0.05 in the reverse MR analyses. The column “driving_SNP” indicated results that failed the leave-on-out test (yes), and the ones that failed the leave-one-out test but were excluded by MR-PRESSO (excluded_by_presso).

Abbreviations: HDL, high-density lipoprotein cholesterol; non-HDL, non-high-density lipoprotein cholesterol; LDL, low-density lipoprotein cholesterol; TC, total cholesterol; TG, triglyceride; snp, single nucleotide polymorphisms; b, causal effect estimate; se, standard error, pl, two-tailed p-value; W, females, M, males.

**Supplementary Table 3.** Mendelian randomization significant results in females, males and in both sexes.

The column “SignificantBysex” highlighted the significant results based on sex in the three different analyses: 1, significant results in females; 2, significant results in males; 3, significant results in both sexes; 4, significant results showing a variance explained < 0.01 in one of the two sexes; 5, no significant results. Two-tailed p-values are reported for all methods analyzed, including IWW, Wald ratio, MR Egger, MR-PRESSO, and Weighted median.

Abbreviations: HDL, high-density lipoprotein cholesterol; non-HDL, non-high-density lipoprotein cholesterol; LDL, low-density lipoprotein cholesterol; TC, total cholesterol; TG, triglyceride; nsnp, number of single nucleotide polymorphisms; b, causal effect estimate; se, standard error, pval, two-tailed p-value, ple, pleiotropy; fdr, false discovery rate; exp_var, variance explained.

**Supplementary Table 4.** Reverse Mendelian randomization results of the significant causal links from Supplementary Table 1.

The table reports the results for the reverse MR analyses, e.g. using the lipids GWAS as exposure and the protein GWAS as outcomes. Two-tailed p-values are reported for all methods analyzed, including IWW, Wald ratio, MR Egger, MR-PRESSO, and Weighted median. The column SignificantBysex highlighted the significant results based on sex: 1, significant results in females; 2, significant results in males; 3_1, significant results in both sexes in the main cist+trans analyses and only significant in females in the bidirectional analyses; 3_2, significant results in both sexes in the main cist+trans analyses and only significant in males in the bidirectional analyses, 3_3, significant results in both sexes in the main cist+trans and in the bidirectional analyses; 4_1, significant results in the main cis+trans analyses showing a variance explained < 0.01 in one of the two sexes and only significant in females in the bidirectional analyses; 5, no significant results.

Abbreviations: HDL, high-density lipoprotein cholesterol; non-HDL, non-high-density lipoprotein cholesterol; LDL, low-density lipoprotein cholesterol; TC, total cholesterol; TG, triglyceride; nsnp, number of single nucleotide polymorphisms; b, causal effect estimate; se, standard error, pval, two-tailed p-value, ple, pleiotropy; fdr, false discovery rate; het, heterozygosity; IV, instrument variable; exp_var, variance explained.

**Supplementary Table 5.** Cochran's Q test results for the significant MR causal relationships.

The column “SignificantBysex” highlighted the significant results based on sex in the three different analyses: 1, significant results in females; 2, significant results in males; 3, significant results in both sexes; 4, significant results showing a variance explained < 0.01 in one of the two sexes; 5, no significant results. The table shows the absolute difference of the betas between males and females (Diff_Beta) and the Cochran's Q test results applied to the weighted betas. Two-tailed p-values are reported for all methods analyzed, including IWW and Wald ratio.

Abbreviations: HDL, high-density lipoprotein cholesterol; non-HDL, non-high-density lipoprotein cholesterol; LDL, low-density lipoprotein cholesterol; TC, total cholesterol; TG, triglyceride; nsnp, number of single nucleotide polymorphisms; b, causal effect estimate; se, standard error, pval, two-tailed p-value, ple, pleiotropy; fdr, false discovery rate; exp_var, variance explained.

**Supplementary Table 6. Multivariable Mendelian randomization significant results in females and males.**

The column “Significant” highlighted the association results: 1, significant results (nominal p value, non-pleotropic and with an F- statistic >10); 0, no significant results. Abbreviations: HDL, high-density lipoprotein cholesterol; non-HDL, non-high-density lipoprotein cholesterol; LDL, low-density lipoprotein cholesterol; TC, total cholesterol; TG, triglyceride; StdError, standard error.

**Supplementary Table 7.** Mendelian randomization results using proteins from the UK Biobank Pharma Proteomic Project (UKB-PPP) as exposures and lipid traits in the Lifelines cohort as outcome.

Two-tailed p-values are reported for all methods analyzed, including IWW, Wald ratio, MR Egger, MR-PRESSO, and Weighted median. A “yes” in the column “Consistency_GLGC_” indicated a replication of the significant result considering the same sex-specific result as in the main analysis, and same direction of causal effect in the whole Lifelines cohort (_all), additionally excluding individuals under lower-lipid medication treatment (_nostatins) and a replication in both Lifelines datasets, the entire cohort and excluding individuals with statins (_Lifelines).

Abbreviations: HDL, high-density lipoprotein cholesterol; non-HDL, non-high-density lipoprotein cholesterol; LDL, low-density lipoprotein cholesterol; TC, total cholesterol; TG, triglyceride; nsnp, number of single nucleotide polymorphisms; b, causal effect estimate; se, standard error, pval, two-tailed p-value, ple, pleiotropy; fdr, false discovery rate; het, heterozygosity; IV, instrument variable; exp_var, variance explained; GLGC, Global Lipid Genetic Consortium.

**Supplementary Table 8.** Sex specific and sex differentiated genes encoding proteins selected in the different analyses with their function, the GWAS catalog annotation, the known drugs currently approved or under investigation, and the target diseases.

**Supplementary Figures**

**Supplementary Figures 1.** Scatter plots performed across all MR methods tested in our study and the leave-one-out sensitivity analysis for the MR carried out. Each black point in the forest plot represents the MR analysis (using the inverse variance weighting method) excluding that particular SNP. The overall analysis including all SNPs is also shown for comparison. **A**. In females. **B**. In males. **C**. In the full dataset.

**Supplementary Figures 2.** Scatter plots performed across all MR methods tested in our study and the leave-one-out sensitivity analysis in the reverse MR analyses. Each black point in the forest plot represents the MR analysis (using the inverse variance weighting method) excluding that particular SNP. The overall analysis including all SNPs is also shown for comparison. **A**. In females. **B**. In males.

**Supplementary Figures 3.** Scatter plots performed across all MR methods tested in our study and the leave-one-out sensitivity analysis in the Lifelines cohort. Each black point in the forest plot represents the MR analysis (using the inverse variance weighting method) excluding that particular SNP. The overall analysis including all SNPs is also shown for comparison. **A1**. Results obtained when using the females-only GWAS. **A2**. Results obtained when using the females-only GWAS but excluding subjects under lower-lipid medication treatment. **B1**. Results obtained when using the males-only GWAS. B**2**. Results obtained when using the males-only GWAS but excluding subjects under lower-lipid medication treatment.

**Supplementary Figure 4. Sex-specific impact of causal relationships after multivariable Mendelian Randomization.**

**A.** The barplot provides a count of proteins selected in each outcome divided by sex. **B.** Venn diagrams showing the number of proteins shared or specific to lipid traits in males and females. For this plot in both panels, only sex-specific relationships are considered.

Abbreviations: HDL, high-density lipoprotein cholesterol; non-HDL, non-high-density lipoprotein cholesterol; LDL, low-density lipoprotein cholesterol; TC, total cholesterol; TG, triglyceride.

**Supplementary Figures 5. Tissue-specific expression enrichment.**

**A. Tissue-specific expression across 54 GTEx v8 tissues.** Tissue enrichment results for genes mapped to the identified sex specific proteins–lipid associations using FUMA’s GENE2FUNC module. Differentially expressed gene sets were tested across 54 individual GTEx v8 tissues, and enrichment was assessed using a two-sided hypergeometric test with Benjamini–Hochberg FDR correction. Bars represent the –log10(FDR) values for each tissue, with red bars indicating significant enrichment (FDR < 0.05) and blue bars indicating non-significant results. **B. General tissue category–level enrichment.** Barplot summarizing expression enrichment across generalized GTEx tissue categories. Bars show –log10(FDR) values, with red bars marking significant enrichment and blue bars marking non-significant categories. Statistical testing and FDR correction were performed as in panel A.
